# COVID-19 pandemic modifies temperature and heat-related illness ambulance transport association in Japan: a nationwide observational study

**DOI:** 10.1186/s12940-021-00808-w

**Published:** 2021-12-02

**Authors:** Xerxes Seposo, Lina Madaniyazi, Chris Fook Sheng Ng, Masahiro Hashizume, Yasushi Honda

**Affiliations:** 1 Nagasaki University School of Tropical Medicine and Global Health, Nagasaki, Japan; 2 Department of Paediatric Infectious Diseases, Institute of Tropical Medicine, Nagasaki University, Nagasaki, Japan; 3 Department of Global Health Policy, Graduate School of Medicine, The University of Tokyo, Tokyo, Japan; 4 Faculty of Health and Sport Sciences, University of Tsukuba, Tsukuba, Japan; 5 National Institute for Environmental Studies, Tsukuba, Japan

**Keywords:** COVID-19, Heat-related illness, Ambulance transport, Effect modification

## Abstract

**Background:**

During the COVID-19 pandemic, several illnesses were reduced. In Japan, heat-related illnesses were reduced by 22% compared to pre-pandemic period. However, it is uncertain as to what has led to this reduction. Here, we model the association of maximum temperature and heat-related illnesses in the 47 Japanese prefectures. We specifically examined how the exposure and lag associations varied before and during the pandemic.

**Methods:**

We obtained the summer-specific, daily heat-related illness ambulance transport (HIAT), exposure variable (maximum temperature) and covariate data from relevant data sources. We utilized a stratified (pre-pandemic and pandemic), two-stage approach. In each stratified group, we estimated the 1) prefecture-level association using a quasi-Poisson regression coupled with a distributed lag non-linear model, which was 2) pooled using a random-effects meta-analysis. The difference between pooled pre-pandemic and pandemic associations was examined across the exposure and the lag dimensions.

**Results:**

A total of 321,655 HIAT cases was recorded in Japan from 2016 to 2020. We found an overall reduction of heat-related risks for HIAT during the pandemic, with a wide range of reduction (10.85 to 57.47%) in the HIAT risk, across exposure levels ranging from 21.69 °C to 36.31 °C. On the contrary, we found an increment in the delayed heat-related risks during the pandemic at Lag 2 (16.33%; 95% CI: 1.00, 33.98%).

**Conclusion:**

This study provides evidence of the impact of COVID-19, particularly on the possible roles of physical interventions and behavioral changes, in modifying the temperature-health association. These findings would have implications on subsequent policies or heat-related warning strategies in light of ongoing or future pandemics.

**Supplementary Information:**

The online version contains supplementary material available at 10.1186/s12940-021-00808-w.

## Introduction

The novel coronavirus which was first reported in Wuhan, China last December 2019 [47], has been spreading globally at unprecedented rate, leading to the virus being declared as a global pandemic by WHO [44]) on 12 March 2020. The clinical disease, COVID-19, associated with the pandemic is caused by the pathogen severe acute respiratory syndrome coronavirus 2 (SARS-CoV-2) [47]. Several countries have introduced either granular (geographically-limited) or nationwide prevention and control measures in order to manage the progression of the disease [17]. In Japan, the government initially rolled out three main strategies; namely: a) early cluster detection and timely response, b) enhancement of early diagnosis and intensive care for severely affected patients, and c) universal healthcare system strengthening alongside behavioral change of the general population [35]. Anchored within these major strategies are policy adaptations and requests highlighted in the “*avoidance of the****3C****’s:****C****losed space,****C****rowded place and****C****lose-contact setting*” strategy issued on February 2020, which specifically encourages social distancing, mask donning and indoor space ventilation [18].

Apart from managing the COVID-19 pandemic, Japan faces yet another seasonal threat due to extreme heat during summer. The country has since documented record-breaking increase in heat-related illness incidence in recent years [19]. The main culprit for the increase in heat-related illnesses is the exposure to extreme heat [36], amplified by the changing climate. In response to this looming threat in the time of the pandemic, the Ministry of Health, Labor and Welfare (MHLW) issued guidelines, dated 4 May 2020, which was entitled and loosely translated to “*Heatstroke prevention in “a new lifestyle” during the COVID-19 pandemic*”, elaborating on how to prevent heatstroke while maintaining the 3Cs [32]. The guidelines explicitly cautioned about the increase in the possibility of heat-related illness risk during the pandemic, particularly with the use of protective face masks (PFM). The stern caution stems from several studies which have noted that PFM use may negatively impact body thermoregulation through increased thermal stress [28, 40] and thus increasing the risk of developing heat-related illnesses.

Contrary to expectations, a recent study observed that heat-related illness ambulance transport (HIAT) decreased during the pandemic by 22% [95% Confidence Interval (CI): 18 – 25%] [16]. The authors noted the role of mobility restrictions as well as remote working in decreasing the exposure of the population to heat, and thus the reduction in HIAT. On the other hand, the observed reduction may also be due to the changes in the exposure-response function, which, most of the time, leads to changes in the health risk/burden. With just little more than a year into the pandemic, evidence regarding the impact of the COVID-19 pandemic on temperature and heat illness association is scarce, and thus making it challenging to attribute whether the changes observed in HIAT was indeed due to the changes in the exposure-health association or otherwise. The determination of how the pandemic impacted the temperature-health outcome associations would help health managers understand which mechanisms may have been linked with these changes and at the same time will serve as a guide in crafting evidence-informed policies in relation to heat-related risk management/strategies in light of ongoing and future pandemics. In this study, we examined the impact of COVID-19 pandemic on the temperature-HIAT associations in Japan.

## Methods

### Data source

Daily summer-specific HIAT data of 47 Japanese prefectures from June to September of 2016 to 2020 were obtained from the Fire and Disaster Management Agency (FDMA) database [10]. Heat-related illness diagnoses are coded using the International Classification of Diseases (ICD) 10. Specifically, the following diagnoses are reported and aggregated as heat-related illness: “heatstroke and sun stroke” (T67.0), “heat syncope” (T67.1), “heat cramp” (T67.2), “heat exhaustion, anhidrotic” (T67.3), “heat fatigue, unspecified” (T67.5), “heat fatigue, transient” (T67.6), “heat edema” (T67.7), and “other effects of heat and light” (T67.8) [23]. Same period maximum temperature (in degrees Celsius; °C) and relative humidity (in %) were obtained from the Japan Meteorological Agency [24]. Pre-pandemic period was from 2016 to 2019, while the pandemic period was in 2020. All (anonymized and aggregated) data used in this analysis were obtained from open-source databases and did not qualify for any ethical approval certification.

### Statistical analysis

We utilized a two-stage analysis in estimating the prefecture-specific and pooled effect modified associations. In the first-stage analysis, we separately modelled all-period and period-specific associations. In both modeling specifications, HIAT was assumed to follow a quasi-Poisson distribution, accounting for overdispersion, per prefecture. Covariates of relative humidity, day of the week, holiday, month, date, year and day of the season (*dos*), based on previous literature [1, 14], were adjusted in the model. All-period association is parameterized as shown in Eq. 1.

1 $${\displaystyle \begin{array}{c}{y}_{i,t}\sim quasiPoisson\\ {}{y}_{i,t}\sim \alpha +c{b}_{maxtemp}+ ns\left( rhave, df=4\right)+ ns\left( dos, df=4\right): factor(year)+ date+ dow+ hod+\varepsilon \end{array}}$$

HIAT from prefecture (*i*) and time (*t*) is modeled with the intercept (*α*); *cb*_*maxtemp*_ is a cross-basis term of maximum temperature; *rhave* represents relative humidity with 4 degrees of freedom (df); *dos* stands for day of the season with 4 df per year, which we allowed to vary by year [14, 26]; linear term for *date*; *dow* is day of the week; *hod* is public holiday; *ε* is the error term. The cross-basis term implemented via a distributed lag non-linear model (DLNM), models the bi-dimensional associations of both the exposure-response and lagged dimensions [13]. The cross-basis term was specified with a quadratic B-spline for the exposure-response dimension with two internal knots placed at the 50th and 90th percentiles of location-specific summer temperature distributions, and a natural cubic B-spline for the lag dimension with an intercept and two equally-spaced internal knots in the log scale. Lag dimension results reflect the risks at the 99th temperature percentile compared to the minimum risk temperature (MRT); the temperature whereby the risk is assumed to be the lowest; in this study it was determined to be at the 1st percentile, which was at 20.9 °C. Due to the immediate nature of HIAT, we set the lag to 5 days. In the period-specific associations, we employed a stratified analysis with pre-pandemic association covering the period from 2016 to 2019 and pandemic association on 2020. The same modeling parameterizations (in Eq. 1) were utilized for the stratified analysis.

In the second stage analysis, prefecture-specific estimates were pooled via random effects meta-analysis with a multivariate meta-regression models of the first-stage coefficients [11, 12]. Here, meta-regression models adjusted for prefecture-specific average maximum temperature and maximum temperature range. Subsequent fitted multivariate meta-regression models were used to estimate the best linear unbiased predictor (BLUP) for the overall cumulative exposure-response in each prefecture. In brief, BLUP allows prefectures with relatively low number of daily HIAT data to utilize the information from larger prefectures sharing similar characteristics [11]. Nationwide pre-pandemic and pandemic period associations, for both exposure and lag dimensions, were derived through the second stage analysis via the R package “mvmeta”. We further examined the difference between pre-pandemic and pandemic associations using a test of interaction [2] and estimated the ratio of relative risks (RRR) [2, 3], along the exposure and lag dimensions. The test of interaction determines whether there is statistical difference between the periods, whereas the subsequent derived RRR estimates the magnitude of the changes in the relative risk by comparing pandemic risk to the baseline (pre-pandemic risk). We followed STROBE reporting guideline for retrospective observational study [43]. All relevant data management and statistical analyses were done using R Statistical programming [39].

### Sensitivity analyses

We also implemented several sensitivity analyses for both all-period and period-specific associations in either exposure or lag dimensions. Specifically, we examined the robustness of the estimates, in terms of the influence of highly-populated locations, i.e. Tokyo and Osaka, on the shape of exposure-response and lag association, by leaving one prefecture out and subsequently running the earlier modeling steps (in Figs. S6 and S7). Furthermore, we have also included the prefecture-specific all-period and period-specific lag and exposure dimensions in the Supplementary Materials (in Figs. S2, S3, S4 and S5) for more detailed appraisal of the prefecture-specific and pooled results. We have also varied the number of internal knots in the exposure dimension and lag dimensions (as shown in Fig. S8) to evaluate its influence on the maximum temperature-HIAT association, in both pre-pandemic and pandemic periods.

## Results

A total of 321,655 HIAT cases was recorded across the country from 2016 to 2020, with most cases occurring in highly populated metropolitan locations of Tokyo (*n* = 25,081) and Osaka (*n* = 23,760) (summarized in Table S1). We observe a discernable gradient of summer-specific maximum temperature (in Fig. S1) with northern locations experiencing milder summers compared to southern locations. In pre-pandemic period, HIAT average daily cases started to rise from the month of June (*n* = 2.9), peaks in the months of July (*n* = 19.9) and August (*n* = 18.2) and drops thereafter in September (*n* = 3.3). However, during the pandemic, we observed a delay in the peak of HIAT cases in August (*n* = 29.6), which recorded 10 additional HIAT cases than pre-pandemic period (in Table 1). In either pre-pandemic or pandemic periods, the highest recorded maximum temperature was in August, whereas for relative humidity it was recorded in July. Fitted lines in the scatterplot (in Fig. S1B) suggest that the association may have been attenuated during the pandemic (in red), with an apparent difference particularly in the high temperature extremes.

**Table 1:** Nationwide summary statistics by period

Variables	Pre-COVID-19 pandemic period (2016-2019)					COVID-19 pandemic period (2020)				
	June	July	August	September	Summer period	June	July	August	September	Summer period
HSAD (*n*)	2.9 (4.8)	19.9 (33.1)	18.2 (27.8)	3.3 (8.1)	11.2 (23. 7)	4.5 (6.2)	5.8 (9.7)	29.6 (37.2)	5.0 (9.7)	11.3 (22.8)
Maximum Temperature (°C)	26.5 (3.4)	30.9 (3.6)	32.3 (3.3)	27.9 (3.5)	29. 5 (4.2)	27.9 (3.2)	28.2 (3.1)	33.8 (2.7)	28.6 (3.8)	29.6 (4.0)
Relative Humidity (%)	73.7 (12.3)	77.0 (9.1)	73.6 (9.6)	76.6 (10.3)	75.3 (10.5)	75.6 (11.6)	84.5 (7.9)	73.3 (7.4)	76.2 (9.9)	77.4 (10.3)

Values in the cells indicate the mean and the standard deviation enclosed in parentheses

*HSAD* Heatstroke Ambulance Dispatch, *n* Number/count, *°C* Degrees Celsius, *%* Percent

**Fig. 1:**
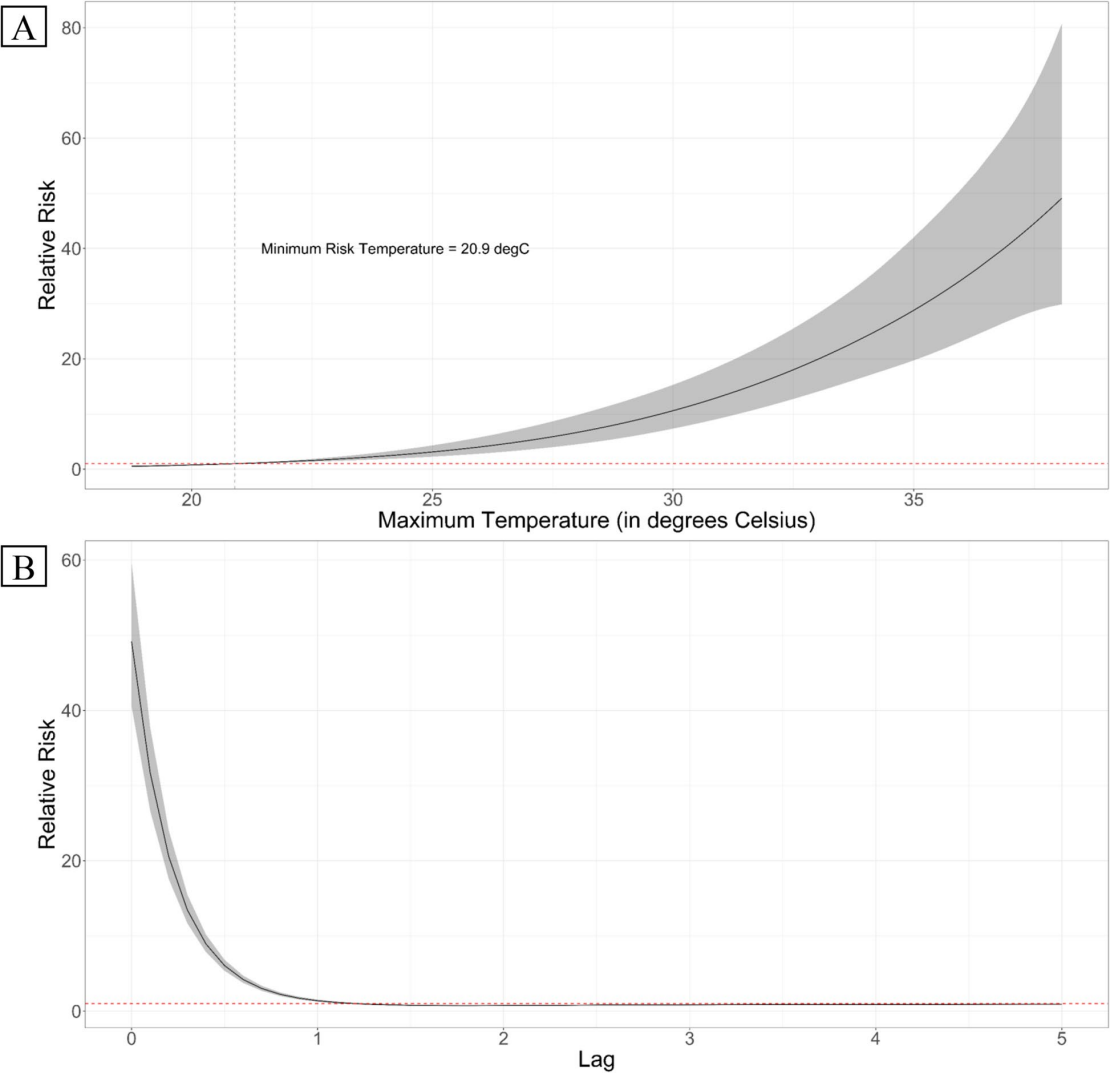
Pooled all-period exposure-response (**A**) and lag (**B**) associations. Centered at the minimum risk temperature (at 20.9 °C; horizontal dotted grey line), the heat-related relative risks increase with increasing temperature (**A**). Red horizontal dotted line represents the null association. Exponential solid line depicts the central estimate of the either exposure-response (**A**) or lag (**B**) associations, with their respective confidence interval (grey-shaded areas)

In the whole study period from 2016 to 2020, shown in Fig. 1A, we observe an exponential increase in the HIAT risk beyond the MRT at 20.9 °C. The gradual increase from the MRT is followed by the steep increase in the risk beyond 30 °C. Whereas, in the lag dimension (in Fig. 1B), same-day maximum temperature exposure was associated with the highest HIAT risk with an RR of 49.2 [95% Confidence Interval (CI): 40.5, 59.6].

**Fig. 2:**
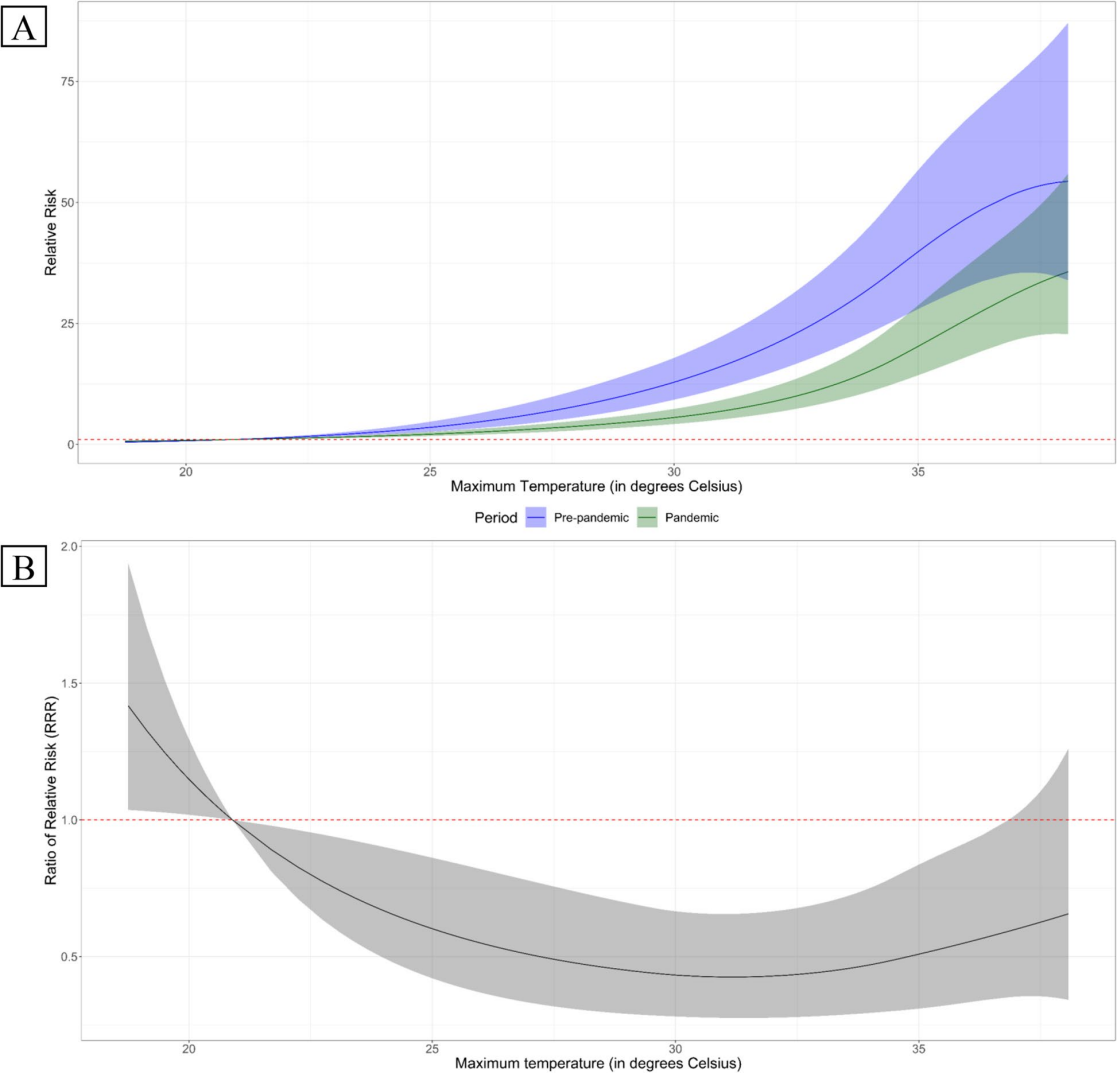
Pooled period-specific exposure-response associations (**A**) and ratio of relative risk (**B**). **A** Pre-pandemic risk (green) and pandemic risks (blue) depicted with their respective central estimates (solid lines) and confidence intervals (color-specific shaded areas). **B** Central estimates of the ratio of relative risks (RRR) in black solid line and its corresponding confidence interval (grey-shaded area)

Further examination revealed an overall reduction in the exposure-response association during the pandemic (in Fig. 2A). Statistical difference between pre-pandemic and pandemic RRs, represented by the RRR in Fig. 2B, was observed across the exposure range. While not visually discernable, risks were slightly higher during the pandemic in the range of temperatures below the MRT. However, beyond the MRT, the risk reduction widened as temperature increased, which waned towards the extreme upper end of the exposure range. The percent reduction ranged from 10.9 to 57.5% between 21.7 °C to 36.3 °C, with the maximum percent reduction at 31.2 °C (57.5%; 95% CI: 34.4, 72.4%). It is notable that the pre-pandemic and pandemic exposure-response associations have similar risk patterns.

**Fig. 3:**
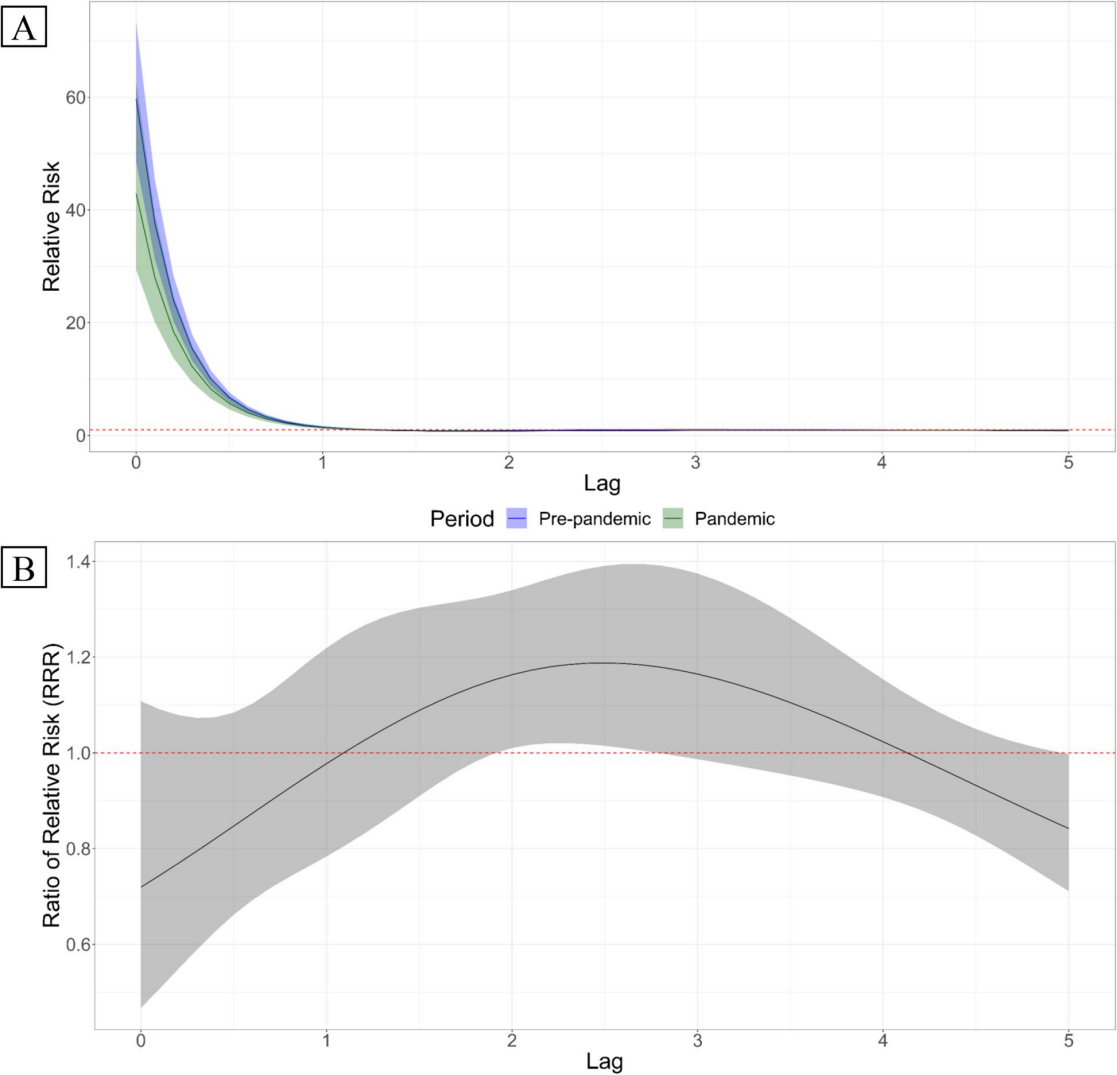
Pooled period-specific lag associations (**A**) and ratio of relative risk (**B**). **A** Pre-pandemic risk (green) and pandemic risks (blue) depicted with their respective central estimates (solid lines) and confidence intervals (color-specific shaded areas). **B** Central estimates of the ratio of relative risks (RRR) in black solid line and its corresponding confidence interval (grey-shaded area)

The lag associations in either periods were comparatively similar with both periods exhibiting same-day maximal risk (in Fig. 3A), which is followed by a sudden drop in the risk at Lag 1 and remains to hover near the null association thereafter. Pre-pandemic lag-specific association at Lag 0 was reduced though statistically not significant, by 28.1% (95% CI: − 53.3, 10.8%) during the pandemic. While similarly visually not discernable, we observed a statistical difference between pre-pandemic and pandemic risk at Lag 2 (in Fig. 3B), with the delayed heat risks to be higher during the pandemic at 16.3% (95% CI: 1.0, 34.0%) than pre-pandemic.

## Discussion

In this study, we found that the COVID-19 pandemic modified both the HIAT exposure-response and lag associations in Japan. An overall reduction in the HIAT risk was generally observed during the COVID-19 pandemic, which may partially be due to the attenuation of both the exposure-response and immediate lag associations. Aside from the reduction, notable risk increments were also apparent in the delayed lag associations. Several mechanisms may have contributed to the reduction or increment of HIAT risk during the pandemic. Firstly, we draw comparison on how these observed changes resemble the impact of large-scale events on heat-related health risks in a more familiar concept of heat-health plans. In addition, we subsequently examined how these changes reflect the role of multi-sectoral policies as well as behavioral changes on the reduction and increment of heat-related health risks.

In the absence of true comparison, we presuppose that the COVID-19 pandemic setting serves as a community-level effect modifier, which share similarities to a heat-health plan. Though not entirely the same, the characterization of preventive measures within a heat wave warning system and measures against COVID-19, which, in part, affect the behaviors of people towards exposures, may be assumed to share the same concept of collective consciousness of risk aversion [30]. In Ahmedabad, India, Hess et al. [20] observed a substantial reduction in the heat-related risk after the implementation of a heat action plan (HAP) in response to the 2010 heat wave event. The authors noted an after-to-before HAP unlagged mortality incidence rate ratio (IRR) of 0.95 (95% CI: 0.93–1.22) and 0.73 (95% CI: 0.29–1.81) for maximum temperatures of over 40 °C and 45 °C, respectively. Here, the authors noted that regardless of the cut off, of either over 40 °C or 45 °C, the reductions in the IRR are still discernable. On the other hand, Martínez-Solanas and Basagaña [29] shares a similar observation, whereby the authors observed a weak to non-significant difference between the overlapping effects estimates of pre-HAP (percent change = 28.0; 95% CI: 22.4 – 33.9%) and post-HAP (percent change = 24.9; 95% CI: 21.4 – 28.6%) periods of high temperature effects in the 50 provinces of Spain. The reduction of heat-related risks due to heat-health plans has a resemblance, albeit different, on how COVID-19 attenuated temperature-HIAT association. Here, a population’s response to the risks is influenced mainly by both physical and behavioral factors [21]. The direction of the changes in the risks would also vary depending on the response. However, in this study, we observed an apparent and consistent reduction in the heat-related risks for both exposure-response (Fig. 2A) and same-day lag dimensions (Fig. 3A). Though it is difficult to draw definitive conclusion on the cause of these reductions, it remains plausible that both physical factors, in terms of policy interventions, accompanied with behavioral changes may have resulted to these changes.

The increased remote working environments as well as the availability of alternative services such as telemedicine, home/food deliveries, may have potentially reduced the ambient exposure of the population [41]. Early in the pandemic, on March 2020, the Japanese government encouraged firms to integrate a work-from-home setting to avoid cluster infections within workplaces. In response, 49.6% of the total number of firms adopted the home working system [34]. The remote work set-up was further complemented with innovations in alternative services (telemedicine and food deliveries) which accompanied the new lifestyle. Health facilities offering tele-medicine in the country [22], has increased during the pandemic, with nearly 10,000 additional clinics offering online services [31]. Similarly, food deliveries have gained traction in the country. In an online survey, approximately 39.7% of 1100 respondents used food delivery, 5% of whom are first-time users [8].

Behavioral changes related to heightened health risk aversion may have also contributed to the substantial decrease in the association [38]. In Japan, a recent study noted that more than 75% of 11,342 respondents, aged 20 to 64 years old, had practiced any form of preventive measures (social distancing, handwashing, coughing etiquette, and immunity fortification) [35]. Though mobility restrictions were not that strict, the response of the population towards these governmental requests have been reciprocated with a significant reduction in trips with the number of inter-prefectural travel halved across the country compared to pre-pandemic conditions [15]. Changes in human behavior during the pandemic is a cognitive response to the immediate threat of COVID-19, which has an indirect effect on the reduction of heat exposure [38]. Specifically, behavioral changes related to time spent indoor/outdoor may be related to the variability in heat exposure [27]. In an online survey conducted from 3 to 25 August 2020 in selected major cities and prefectures in Japan (*n* = 12,872), there was an approximately 13.8% increase in time spent indoors during the pandemic compared to pre-pandemic period, which subsequently corresponded to an 18.8% reduction in outdoor activities across the country [33]. Since HIAT occur mostly in roads (15.6%) and outdoor public areas (12.5%) [16], the increase in time spent indoor may have led to the attenuation of the heat-related health risks during the pandemic.

On the other hand, the pandemic may, in part, have altered the health-seeking behavior of the population. This is quite apparent in the significant difference in Lag 2, whereby pandemic risks were higher compared to the pre-pandemic period. It is plausible that this phenomenon is related to the reduction of same-day risks observed during the pandemic. During the pandemic, several studies have noted the delay of access to medical care [6, 9], with the fear of contracting the disease [5, 45]. In the US, a survey revealed that 41% of the respondents have foregone medical care in the early phase of the pandemic [4]. In another online survey, 12% of 4975 respondents opted to delay or have avoided urgent or emergency medical care [9]. It is reasonable to believe that those populations which have foregone immediate medical services (i.e. hospital/ambulance transport) may have delayed their access to a later period, thus mirroring the reduction in the same-day risk and a subsequent increase in the delayed association.

While we have observed a reduction in the exposure-response association during the pandemic, there was a notable statistically significant delayed lag association at lag 2, with a 16.3% (95% CI: 1.0, 34.0%) increase in the risk of HIAT during the pandemic compared to pre-pandemic period. The increment in the delayed risk during the pandemic period may have potential implications to health service access. In part, this may be loosely related to the delay in accessing health services due to medical care access hesitancy during the pandemic and of other related barriers such as informational/technological resource access [9, 37]. Health service providers and managers would then need to consider these factors and other potential mechanisms in order to overcome the barriers to healthcare access in the pandemic-stricken setting. In terms of heat-related health risk management, access to the appropriate information, such as clinic availability/schedule which provide telemedicine [7] as well as readily-accessible geographical maps of health facilities catering to non-COVID-19 cases [25], may potentially ease up the hesitancy in accessing immediate health care services.

We, however, note several limitations in this study. The ecological nature of the study may not be able to capture the individual-level characteristics, which may possibly explain the changes in the association. Further studies are needed to account for these personal-level data. In the absence of 2019 air pollution data, we were not able to examine the role of air pollution, particularly particulate matter with a diameter less than 2.5 μm in size (PM_2.5_), on the temperature-HIAT associations; a complete data is needed whenever available. However, it would be prudent to assume that the effect of air pollution on temperature-HIAT associations would be minimal, since 1) Japan has a relatively low ambient air pollution level [46] compared to other developed countries, and 2) that this low ambient air pollution may have been further reduced during the pandemic, which was similarly observed across several countries globally [42]. Also, the pandemic period data coverage is limited to 2020. Data supplementation would be necessary whenever available in order to further establish the robustness of the results. In this study, we utilized a simple binary indicator to represent COVID-19 impact. There is merit for other studies to explore other indicators which could represent the magnitude of the pandemic’s impact, i.e. continuous metrics. Nevertheless, we believe that the results provide substantial evidence on the magnitude of temperature-HIAT association reduction or increment during the pandemic. The results can serve as a guidepost for health managers and policymakers in crafting subsequent heat-health advisories as we enter the second year into the pandemic as well as future disease entities with pandemic potential.

## Conclusion

In summary, current evidence suggests that the COVID-19 pandemic has modified temperature-health outcome associations; in this case that of heat-related illness ambulance transports in Japan. These changes have been generally rooted in the physical and behavioral changes which have had transpired. Particularly, the differential impact of COVID-19 pandemic, evident in the variations in the direction of exposure and lag associations, requires an immediate attention and more in-depth examination in order to aid health managers and policymakers in assessing the true extent of the impact of the pandemic as well as provide guidance on subsequent heat-related warnings in light of ongoing and future pandemics.

## Supplementary Information

**Additional file 1: Table S1.** Prefecture-specific HIAT, maximum temperature, and socio-demographic characteristics. **Figure S1.** All-period summer average temperature per prefecture (*panel A*) and the nationwide period-specific scatterplots (*panel B*). **Figure S2.** Prefecture-specific all-period exposure-response association. **Figure S3.** Prefecture-specific all-period lag association. **Figure S4.** Prefecture-specific, pre-pandemic (blue) and pandemic (green) exposure-response associations. **Figure S5.** Prefecture-specific, pre-pandemic (blue) and pandemic (green) lag associations. **Figure S6.** Sensitivity analyses for all-period (*panel A*) and period-specific exposure-response associations (*panel B*). **Figure S7.** Sensitivity analyses for all-period (*panel A*) and period-specific Lag associations (*panel B*). **Figure S8.** Sensitivity analysis of the knot parameterization for exposure (*Panel A*) and lag (*Panel B*) dimensions (in the case of Tokyo). **Figure S9.** Residual distribution accounting for seasonal patterns.

## Data Availability

All relevant data utilized in the study are properly acknowledged in the Methods section are accessible in the respective data source sites.

## References

[1] Achebak H, Devolder D, Ballester J (2018). Heat-related mortality trends under recent climate warming in Spain: a 36-year observational study. PLoS Med.

[2] Altman DG, Bland JM (2003). Interaction revisited: the difference between two estimates. BMJ.

[3] Altman DG, Bland JM (2011). How to obtain the p value from a confidence interval. BMJ.

[4] Anderson KE, McGinty EE, Presskreischer R, Barry CL (2021). Reports of forgone medical care among us adults during the initial phase of the covid-19 pandemic. JAMA Netw Open.

[5] Apisarnthanarak A, Siripraparat C, Apisarnthanarak P, Ullman M, Saengaram P, Leeprechanon N, et al. Patients’ anxiety, fear, and panic related to coronavirus disease 2019 (covid-19) and confidence in hospital infection control policy in outpatient departments: a survey from four Thai hospitals. Infect Control Hosp Epidemiol. 2021;42:1288–90. 10.1017/ice.2020.1240 PMC7573456 33023718

[6] Bhambhvani HP, Rodrigues AJ, Yu JS, Carr JB, Hayden GM (2021). Hospital volumes of 5 medical emergencies in the covid-19 pandemic in 2 us medical centers. JAMA Intern Med.

[7] Budd J, Miller BS, Manning EM, Lampos V, Zhuang M, Edelstein M (2020). Digital technologies in the public-health response to covid-19. Nat Med.

[8] Cross Marketing (2020). Survey on food delivery services and food delivery.

[9] Czeisler M, Marynak K, Clarke KEN, Salah Z, Shakya I, Thierry JAM (2020). Delay or avoidance of medical care because of covid-19-related concerns - united states, june 2020. MMWR Morb Mortal Wkly Rep.

[10] FDMA (2020). Fire and disaster management agency.

[11] Gasparrini A, Armstrong B, Kenward MG (2012). Multivariate meta-analysis for non-linear and other multi-parameter associations. Stat Med.

[12] Gasparrini A, Armstrong B (2013). Reducing and meta-analysing estimates from distributed lag non-linear models. BMC Med Res Methodol.

[13] Gasparrini A, Leone M (2014). Attributable risk from distributed lag models. BMC Med Res Methodol.

[14] Gasparrini A, Guo Y, Hashizume M, Kinney PL, Petkova EP, Lavigne E (2015). Temporal variation in heat-mortality associations: a multicountry study. Environ Health Perspect.

[15] Hara Y, Yamaguchi H (2021). Japanese travel behavior trends and change under covid-19 state-of-emergency declaration: nationwide observation by mobile phone location data. Transp Res Interdiscip Perspect.

[16] Hatakeyama K, Ota J, Takahashi Y, Kawamitsu S, Seposo X (2021). Effect of the covid-19 pandemic on heatstroke-related ambulance dispatch in the 47 prefectures of Japan. Sci Total Environ.

[17] Haug N, Geyrhofer L, Londei A, Dervic E, Desvars-Larrive A, Loreto V (2020). Ranking the effectiveness of worldwide covid-19 government interventions. Nat Hum Behav.

[18] Hayashi M, Yanagi U, Azuma K, Kagi N, Ogata M, Morimoto S (2020). Measures against covid-19 concerning summer indoor environment in Japan. Jpn Archit Rev.

[19] Hayashida K, Shimizu K, Yokota H (2019). Severe heatwave in Japan. Acute Med Surg.

[20] Hess JJ, Lm S, Knowlton K, Saha S, Dutta P, Ganguly P (2018). Building resilience to climate change: pilot evaluation of the impact of india’s first heat action plan on all-cause mortality. J Environ Public Health.

[21] Howe PD, Marlon JR, Wang X, Leiserowitz A (2019). Public perceptions of the health risks of extreme heat across us states, counties, and neighborhoods. Proc Natl Acad Sci.

[22] Ito J, Edirippulige S, Aono T, Armfield NR (2017). The use of telemedicine for delivering healthcare in Japan: systematic review of literature published in japanese and english languages. J Telemed Telecare.

[23] JAAM. Medical practice guidelines for heat stroke. Tokyo: Japanese Association for Acute Medicine; 2015.

[24] JMA. Weather data. Tokyo: Japan Meteorological Agency; 2020.

[25] Kamel Boulos MN, Geraghty EM (2020). Geographical tracking and mapping of coronavirus disease covid-19/severe acute respiratory syndrome coronavirus 2 (sars-cov-2) epidemic and associated events around the world: how 21st century gis technologies are supporting the global fight against outbreaks and epidemics. Int J Health Geogr.

[26] Kim Y, Gasparrini A, Hashizume M, Honda Y, Ng CFS, Armstrong B (2017). Heat-related mortality in Japan after the 2011 Fukushima disaster: an analysis of potential influence of reduced electricity consumption. Environ Health Perspect.

[27] Kuras ER, Richardson MB, Calkins MM, Ebi KL, Hess JJ, Kintziger KW (2017). Opportunities and challenges for personal heat exposure research. Environ Health Perspect.

[28] Lin Y-C, Chen C-P (2019). Thermoregulation and thermal sensation in response to wearing tight-fitting respirators and exercising in hot-and-humid indoor environment. Build Environ.

[29] Martínez-Solanas È, Basagaña X (2019). Temporal changes in temperature-related mortality in Spain and effect of the implementation of a heat health prevention plan. Environ Res.

[30] Matthies F, Menne B (2009). Prevention and management of health hazards related to heatwaves. Int J Circumpolar Health.

[31] MHLW. 2020a. Online medical care in light of the spread of new coronavirus infections. Tokyo: Ministry of Health, Labour and Welfare; 2020.

[32] MHLW. 2020b. A note on preventive actions against heat stroke in 2020: prevention of heat stroke in a “new lifestyle” in anticipation of a new coronavirus. Tokyo: Ministry of Health, Labour and Welfare; 2020.

[33] MILT. First nationational questionnaire survey on individuals’ 24-hour usage at three points in time: before the outbreak of h1n1 corona, during the declaration of a state of emergency, and after the declaration was lifted (preliminary report). Tokyo: Ministry of Land, Infrastructure, Transport and Tourism; 2020.

[34] Morikawa M. Productivity of working from home during the covid-19 pandemic: evidence from an employee survey. (Discussion papers). 20073. Tokyo: Research Institute of Economy, Trade and Industry (RIETI); 2020.

[35] Muto K, Yamamoto I, Nagasu M, Tanaka M, Wada K (2020). Japanese citizens’ behavioral changes and preparedness against covid-19: an online survey during the early phase of the pandemic. PLoS One.

[36] Ng CF, Ueda K, Ono M, Nitta H, Takami A (2014). Characterizing the effect of summer temperature on heatstroke-related emergency ambulance dispatches in the Kanto area of Japan. Int J Biometeorol.

[37] Núñez A, Sreeganga SD, Ramaprasad A (2021). Access to healthcare during covid-19. Int J Environ Res Public Health.

[38] Parady G, Taniguchi A, Takami K (2020). Travel behavior changes during the covid-19 pandemic in Japan: analyzing the effects of risk perception and social influence on going-out self-restriction. Transp Res Interdiscip Perspect.

[39] R Core Team (2013). R: a language and environment for statistical computing.

[40] Roberge RJ, Kim J-H, Coca A (2011). Protective facemask impact on human thermoregulation: an overview. Ann Occup Hyg.

[41] Tamura Y, Takeyasu R, Furukawa A, Takada H, Takechi M, Taniguchi H (2020). How covid-19 affected the introduction of telemedicine and patient reported outcomes among patients with pulmonary hypertension ― a report from a referral center in Japan. Circ Rep.

[42] Venter ZS, Aunan K, Chowdhury S, Lelieveld J (2020). Covid-19 lockdowns cause global air pollution declines. Proc Natl Acad Sci U S A.

[43] von Elm E, Altman DG, Egger M, Pocock SJ, Gøtzsche PC, Vandenbroucke JP (2007). The strengthening the reporting of observational studies in epidemiology (strobe) statement: guidelines for reporting observational studies. Ann Intern Med.

[44] WHO (2020). Who director-general’s opening remarks at the mission briefing on covid-19 - 12 March 2020.

[45] Wong LE, Hawkins JE, Langness S, Murrell KL, Iris P, Sammann A. Where are all the patients? Addressing covid-19 fear to encourage sick patients to seek emergency care. NEJM Catal Innov Care Deliv. 2020. 10.1056/CAT.1020.0193.

[46] Zhao B, Johnston FH, Salimi F, Kurabayashi M, Negishi K (2020). Short-term exposure to ambient fine particulate matter and out-of-hospital cardiac arrest: a nationwide case-crossover study in Japan. Lancet Planet Health.

[47] Zhu N, Zhang D, Wang W, Li X, Yang B, Song J (2020). A novel coronavirus from patients with pneumonia in China, 2019. N Engl J Med.

