# Supplementary material for: COVID-19 pandemic modifies temperature and heat-related illness ambulance transport association in Japan: a nationwide observational study
Source: Environ Health. 2021 Dec 2;20:122. doi: 10.1186/s12940-021-00808-w (PMC8637525; doi:10.1186/s12940-021-00808-w)
Supplement: Supplementary file 1 — Additional file 1: Table S1. Prefecture-specific HIAT, maximum temperature, and socio-demographic characteristics. Figure S1. All-period summer average temperature per prefecture (panel A) and the nationwide period-specific scatterplots (panel B). Figure S2. Prefecture-specific all-period exposure-response association. Figure S3. Prefecture-specific all-period lag association. Figure S4. Prefecture-specific, pre-pandemic (blue) and pandemic (green) exposure-response associations. Figure S5. Prefecture-specific, pre-pandemic (blue) and pandemic (green) lag associations. Figure S6. Sensitivity analyses for all-period (panel A) and period-specific exposure-response associations (panel B). Figure S7. Sensitivity analyses for all-period (panel A) and period-specific Lag associations (panel B). Figure S8. Sensitivity analysis of the knot parameterization for exposure (Panel A) and lag (Panel B) dimensions (in the case of Tokyo). Figure S9. Residual distribution accounting for seasonal patterns. [file 12940_2021_808_MOESM1_ESM.pdf]

Supplementary Materials

COVID-19 pandemic modifies temperature and heat-related illness ambulance transport association in Japan: A nationwide observational study

Xerxes Seposo, Lina Madanayazi, Chris Fook Sheng Ng, Masahiro Hashizume and Yasushi Honda

Table of Contents

Table S1. Prefecture-specific HIAT, maximum temperature, and socio-demographic characteristics 2

Figure S1. All-period summer average temperature per prefecture (*panel A*) and the nationwide period-specific scatterplots (*panel B*) 5

Figure S2. Prefecture-specific all-period exposure-response association............6

Figure S3. Prefecture-specific all-period lag association 7

Figure S4. Prefecture-specific, pre-pandemic (blue) and pandemic (green) exposure-response associations............8

Figure S5. Prefecture-specific, pre-pandemic (blue) and pandemic (green) lag associations............9

Figure S6. Sensitivity analyses for all-period (*panel A*) and period-specific exposure-response associations (*panel B*) 10

Figure S7. Sensitivity analyses for all-period (*panel A*) and period-specific Lag associations (*panel B*)............12

Figure S8. Sensitivity analysis of the knot parameterization for exposure (*Panel A*) and lag (*Panel B*) dimensions (in the case of Tokyo)............14

Figure S9. Residual distribution accounting for seasonal patterns 15

Table S1. Prefecture-specific HIAT, maximum temperature, and socio-demographic characteristics

Prefecture	HIAT	Study period average (*of maximum temperature; ℃*)	Study period interquartile range (*℃*)	2018 Population (*per 100,000 population*)	Land area (*in* km^2)	2011 Gross Prefectural Product (*in 1,000,000 yen*)
Hokkaido	5746	24.43115	5.2	52.86	78420.77	19018098
Aomori	1993	25.71115	5	12.63	9645.65	4580259
Iwate	2733	26.55197	6	12.41	15275.01	4674256
Miyagi	4847	26.51705	6.2	23.16	7282.23	9475481
Akita	2460	26.98967	5.675	9.81	11637.52	3451335
Yamagata	2712	28.3	6.1	10.9	9323.15	4039808
Fukushima	5505	28.49443	7	18.64	13783.9	7917871
Ibaraki	7621	28.07098	5.975	28.77	6097.33	13056738
Tochigi	4658	28.79344	6.5	19.46	6408.09	8958397
Gunma	6150	29.52738	6.3	19.52	6362.28	8528499
Saitama	19058	29.99377	7.175	73.3	3797.75	22689675
Chiba	14126	28.88574	5.9	62.55	5157.61	20391622
Tokyo	25081	29.13754	6.075	138.22	2193.96	104470026
Kanagawa	15181	28.87525	6.1	91.77	2416.16	34609343
Niigata	6182	28.0318	5.575	22.46	12584.23	8883972
Toyama	2229	29.0577	6.275	10.5	4247.61	4566284
Ishikawa	3045	29.05836	5.8	11.43	4186.05	4623028

Fukui	1953	29.83098	6.075	7.74	4190.52	3211131
Yamanashi	2228	30.84361	5.9	8.17	4465.27	3365637
Nagano	4478	28.75148	6.5	20.63	13561.56	8272256
Gifu	6314	31.0018	5.675	19.97	10621.29	7621798
Shizuoka	8592	29.57459	4.9	36.59	7777.35	17044389
Aichi	20692	30.86672	5.6	75.37	5172.96	39409405
Mie	5841	29.37262	5.2	17.91	5774.42	8220907
Shiga	3547	29.53803	5.975	14.12	4017.38	6381694
Kyoto	8418	31.25557	6.4	25.91	4612.2	10487555
Osaka	23760	31.0523	5.95	88.13	1905.29	38994994
Hyogo	15753	29.83262	5	54.84	8400.95	20937780
Nara	4652	30.74607	6.2	13.39	3690.94	3650718
Wakayama	3344	30.32311	4.9	9.35	4724.65	3676471
Tottori	2099	30.13787	6.1	5.6	3507.14	1864072
Shimane	2138	29.13836	6	6.8	6708.07	2520649
Okayama	7473	30.7182	6.175	18.98	7114.33	7681163
Hiroshima	8437	30.2023	5.775	28.17	8479.61	11944686
Yamaguchi	3534	30.66885	6.275	13.7	6112.53	6087533
Tokushima	2341	29.97869	5.275	7.36	4146.75	3071972
Kagawa	3164	30.66049	6.35	9.62	1876.78	3802234
Ehime	4485	30.41262	5.8	13.52	5676.24	5074178
Kochi	2563	30.20361	4.6	7.06	7103.63	2419434
Fukuoka	13182	30.49311	5.9	51.07	4986.51	19144020

Saga	2941	30.94	6.175	8.19	2440.7	2851913
Nagasaki	4295	29.71115	5.275	13.41	4130.9	4566162
Kumamoto	6994	31.12492	5.8	17.57	7409.5	5927626
Oita	3859	29.87393	6.2	11.44	6340.73	4353384
Miyazaki	3848	29.85082	4.5	10.81	7735.32	3683966
Kagoshima	6804	30.95967	4.8	16.14	9187.02	5381809
Okinawa	4599	31.33967	2.3	14.48	2281.05	4281963

*km ≡ kilometer squared; HIAT ≡ heat-related illness ambulance transport; ℃ ≡ degrees Celsius

Figure S1. All-period summer average temperature per prefecture (*panel A*) and the nationwide period-specific scatterplots (*panel B*)

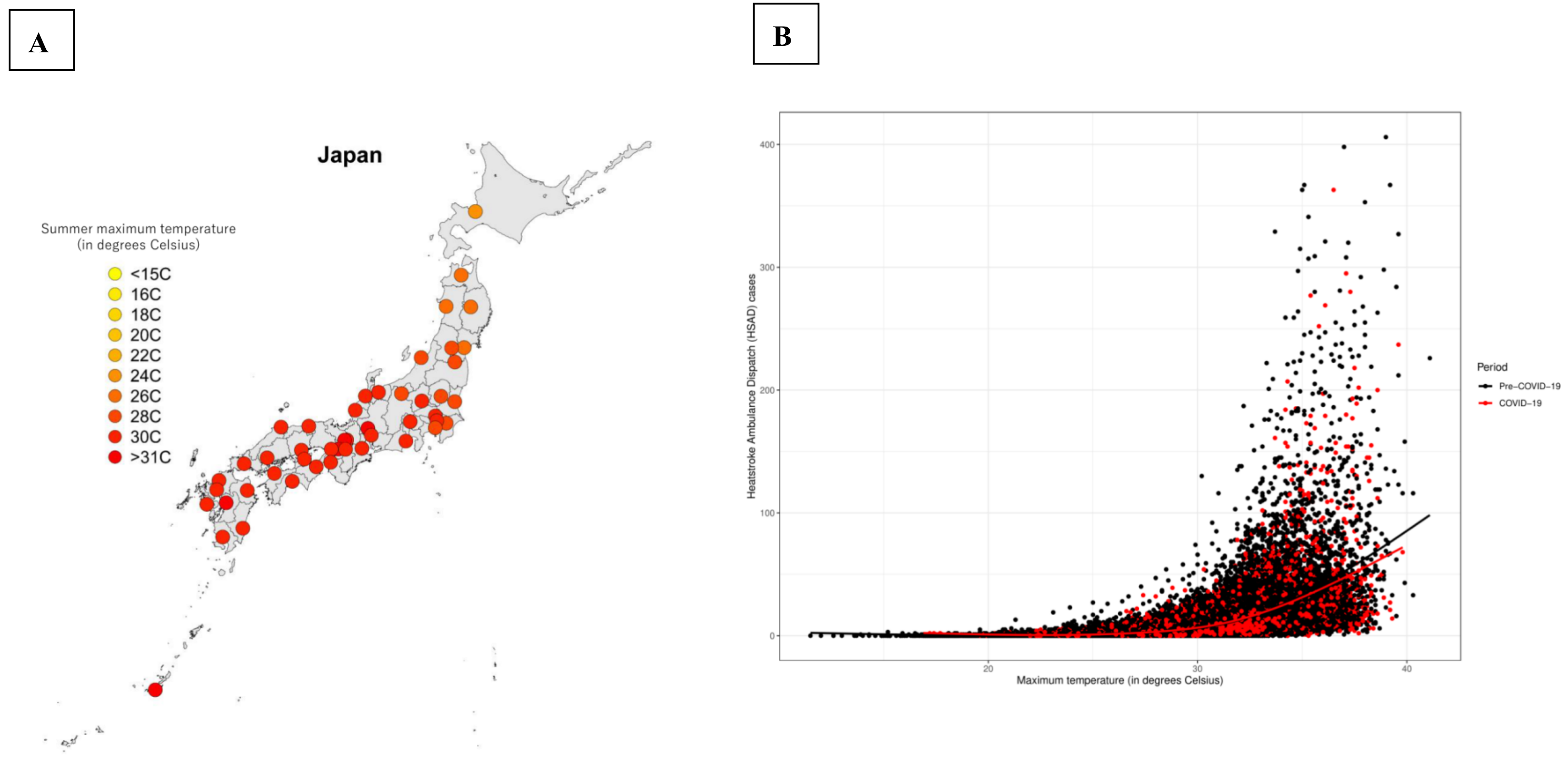

Gradient-colored dots indicate the prefecture specific summer average temperatures from 2016-2020. While in the immediate right panel, pre-pandemic (black colored dots) and pandemic (red dots) scatterplots indicate similarly increasing trends, but with a branching away of the associations (red and black solid lines) in the upper temperature extremes.

Figure S2. Prefecture-specific all-period exposure-response association

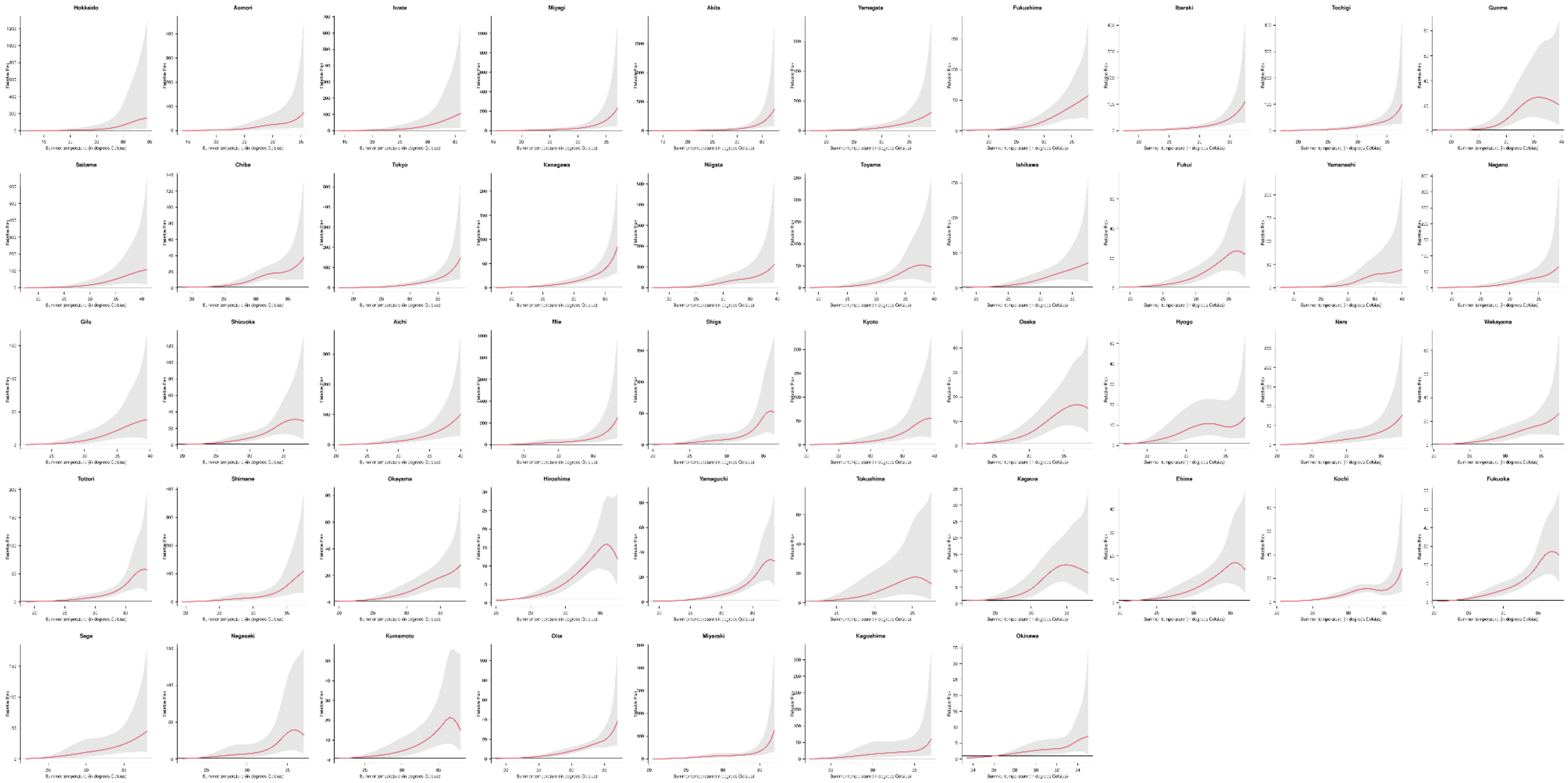

Central estimates are shown in red, solid line, with the corresponding 95% Confidence Interval (grey-shaded areas).

Figure S3. Prefecture-specific all-period lag association

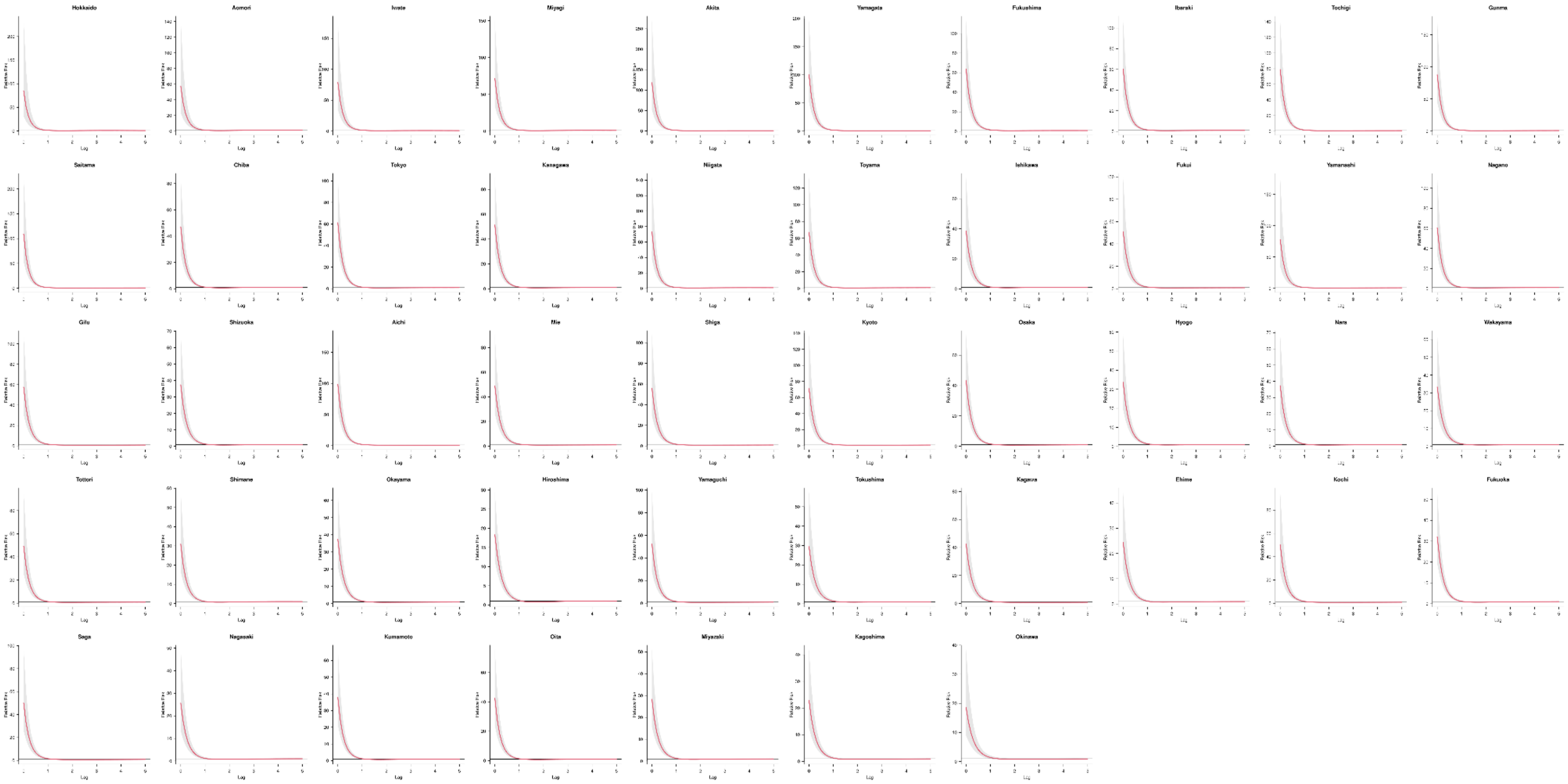

Central estimates are shown in red, solid line, with the corresponding 95% Confidence Interval (grey-shaded areas).

Figure S4. Prefecture-specific, pre-pandemic (blue) and pandemic (green) exposure-response associations

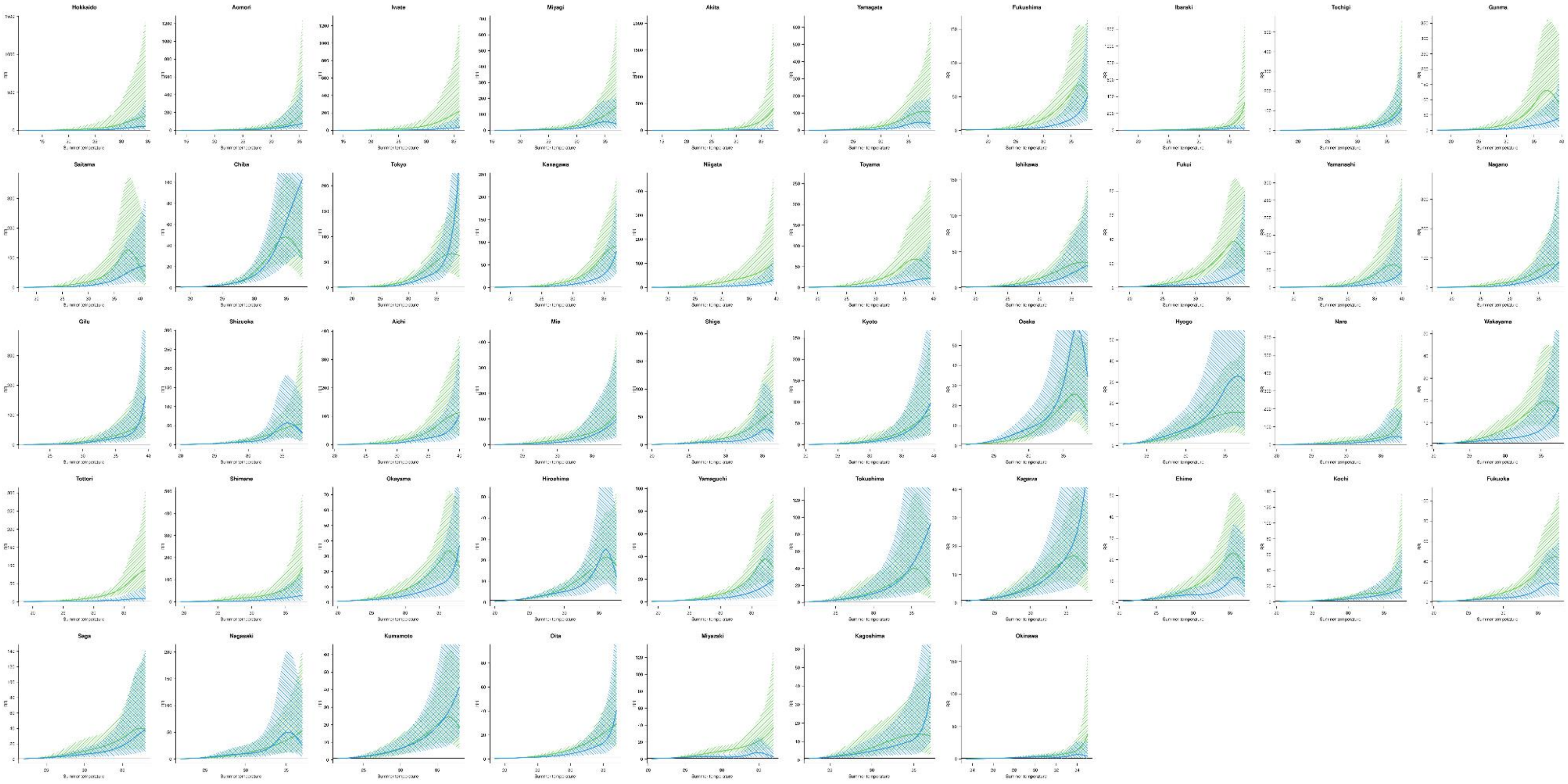

Central estimates for pre-pandemic and pandemic associations are shown in blue and green solid lines, respectively, alongside their corresponding 95% Confidence Interval (color-specific, shaded areas).

Figure S5. Prefecture-specific, pre-pandemic (blue) and pandemic (green) lag associations

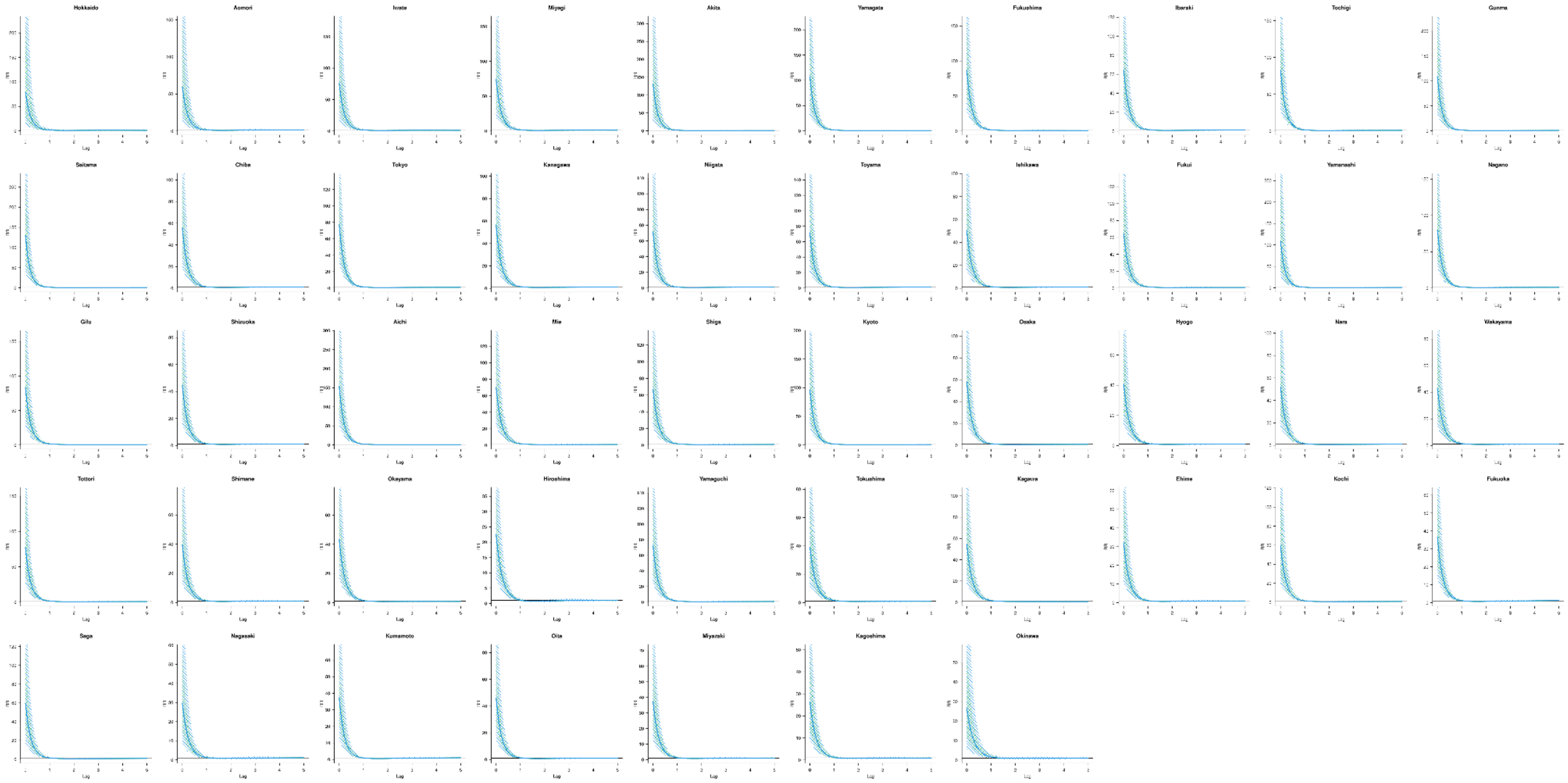

Central estimates for pre-pandemic and pandemic associations are shown in blue and green solid lines, respectively, alongside their corresponding 95% Confidence Interval (color-specific, shaded areas).

Figure S6. Sensitivity analyses for all-period (*panel A*) and period-specific exposure-response associations (*panel B*)

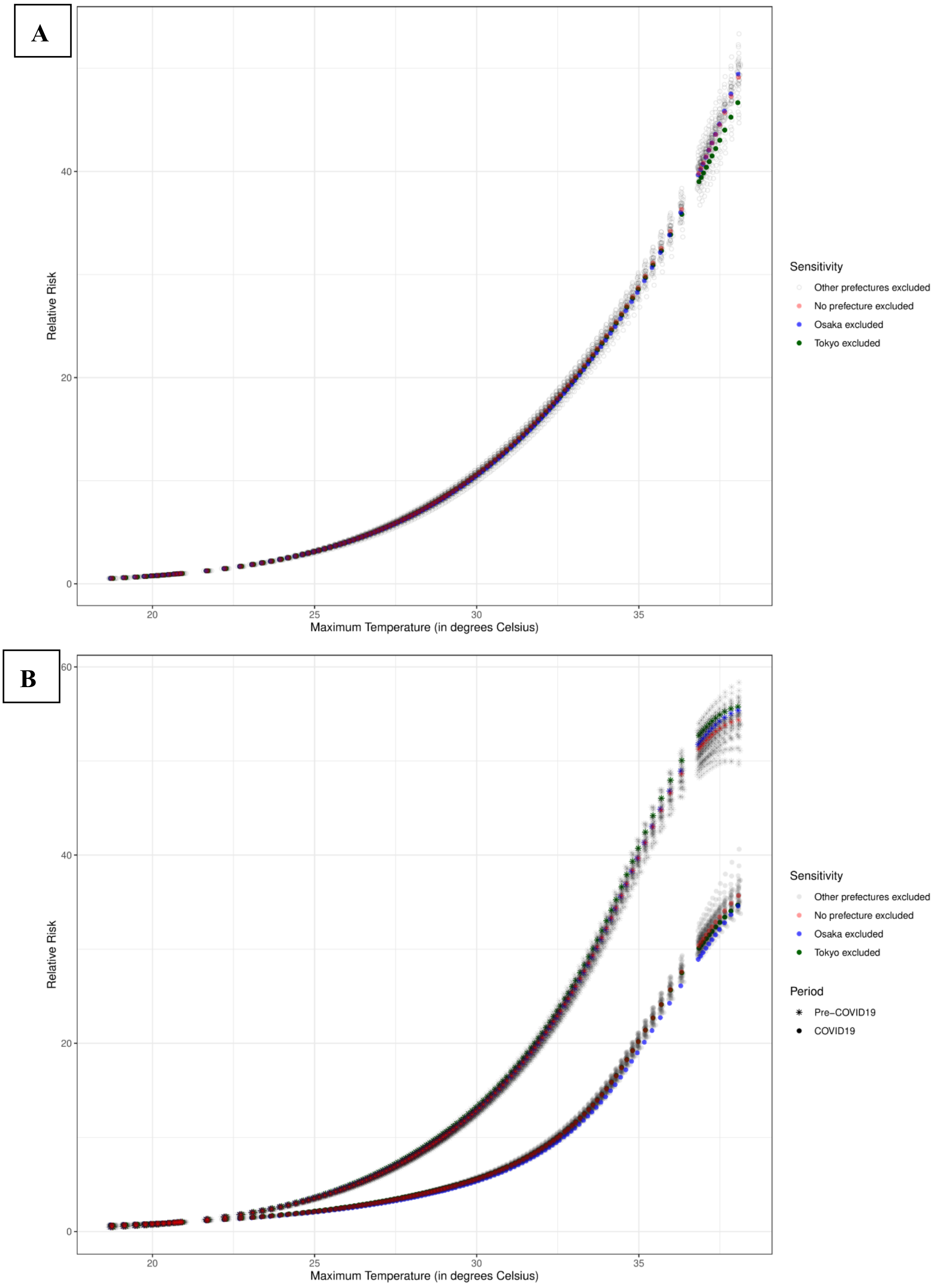

Since the confidence intervals (CIs) are overlapping across each sensitivity analysis, only the central estimates were shown. The pattern of the CIs is similar to that in **Figure 1A**. Similarly centered at 20.9°C for both all-period and period-specific associations. Pre-pandemic associations are depicted with asterisks, whereas pandemic associations are shown in solid dots. Correspondingly, the estimates where major locations of Tokyo and Osaka were excluded are shown in green and blue, respectively, while the other prefecture excluded associations are shown in grey. The estimates in red represent the association whereby all locations were utilized, and none were excluded (essentially **Figure 1A**).

Figure S7. Sensitivity analyses for all-period (*panel A*) and period-specific Lag associations (*panel B*)

A

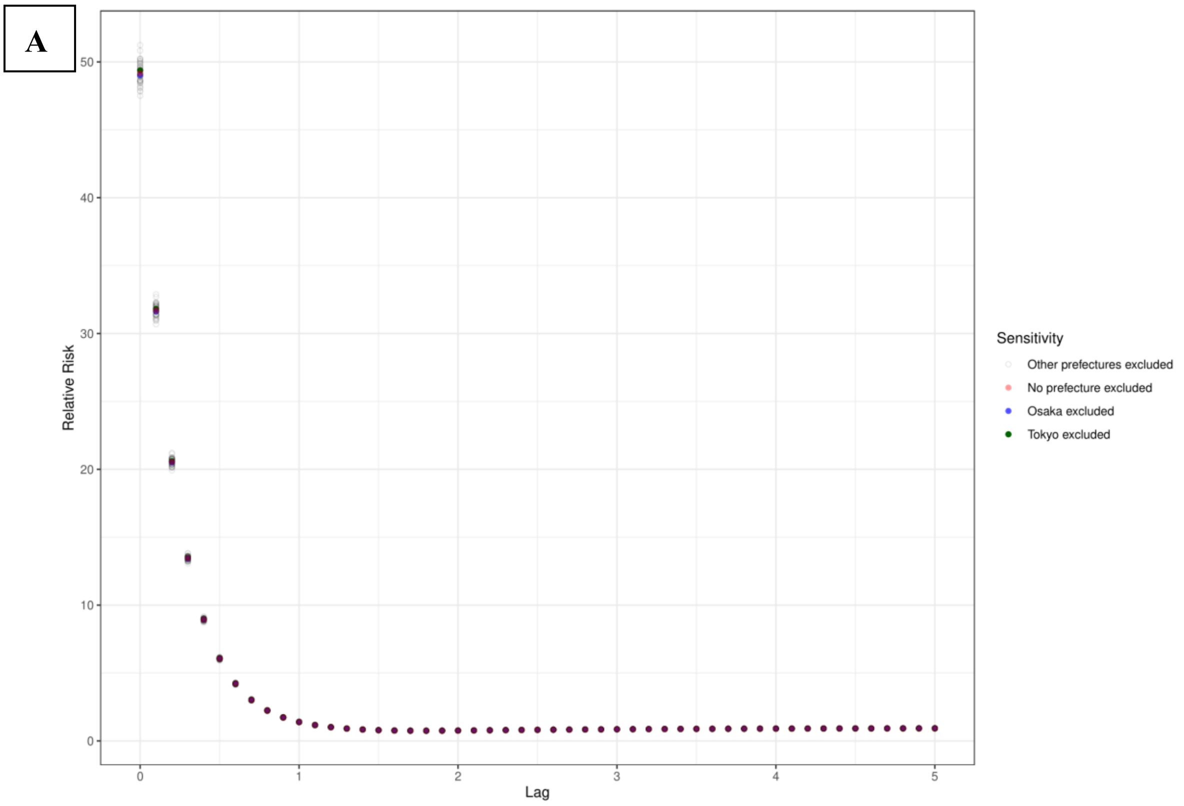

B

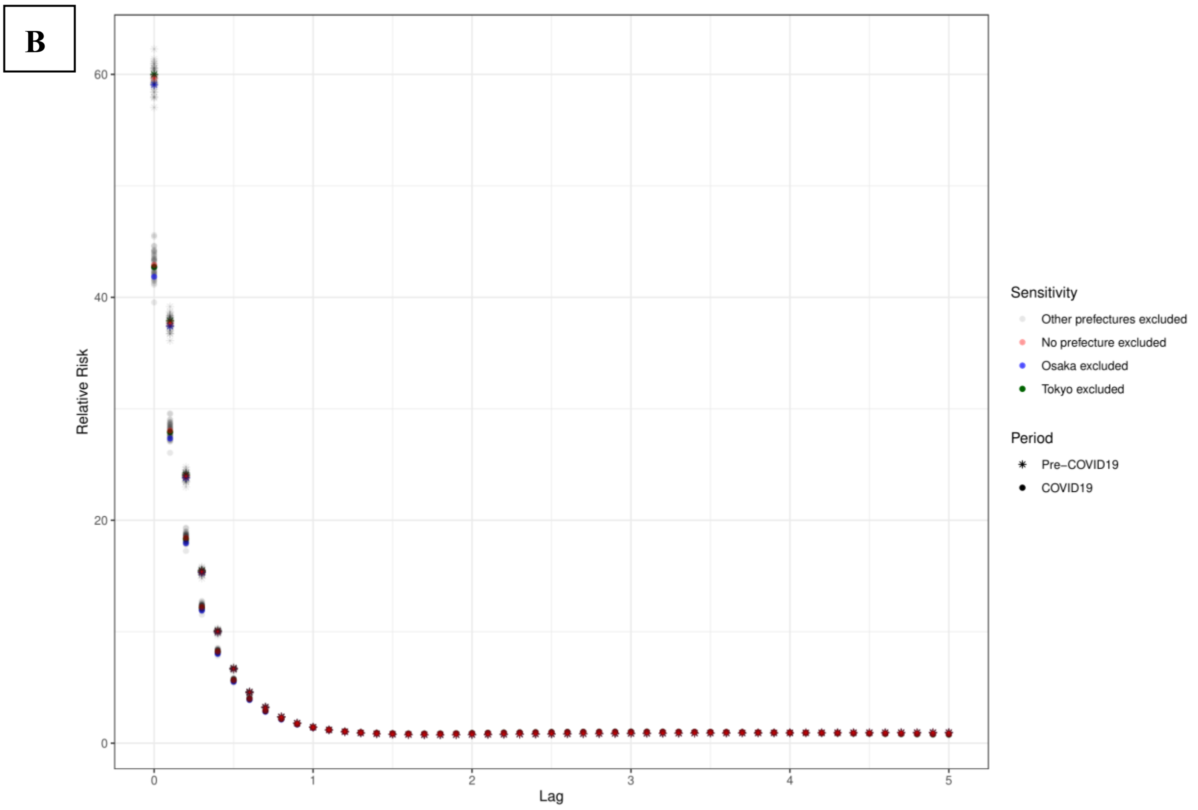

Since the confidence intervals (CIs) are overlapping across each sensitivity analysis, only the central estimates were shown. The pattern of the CIs is similar to that in **Figure 1B**. Similarly centered at 20.9°C for both all-period and period-specific associations. Pre-pandemic associations are depicted with asterisks, whereas pandemic associations are shown in solid dots. Correspondingly, the estimates where major locations of Tokyo and Osaka were excluded are shown in green and blue, respectively, while the other prefecture excluded associations are shown in grey. The estimates in red represent the association whereby all locations were utilized, and none were excluded (essentially **Figure 1B**).

Figure S8. Sensitivity analysis of the knot parameterization for exposure (*Panel A*) and lag (*Panel B*) dimensions (in the case of Tokyo)

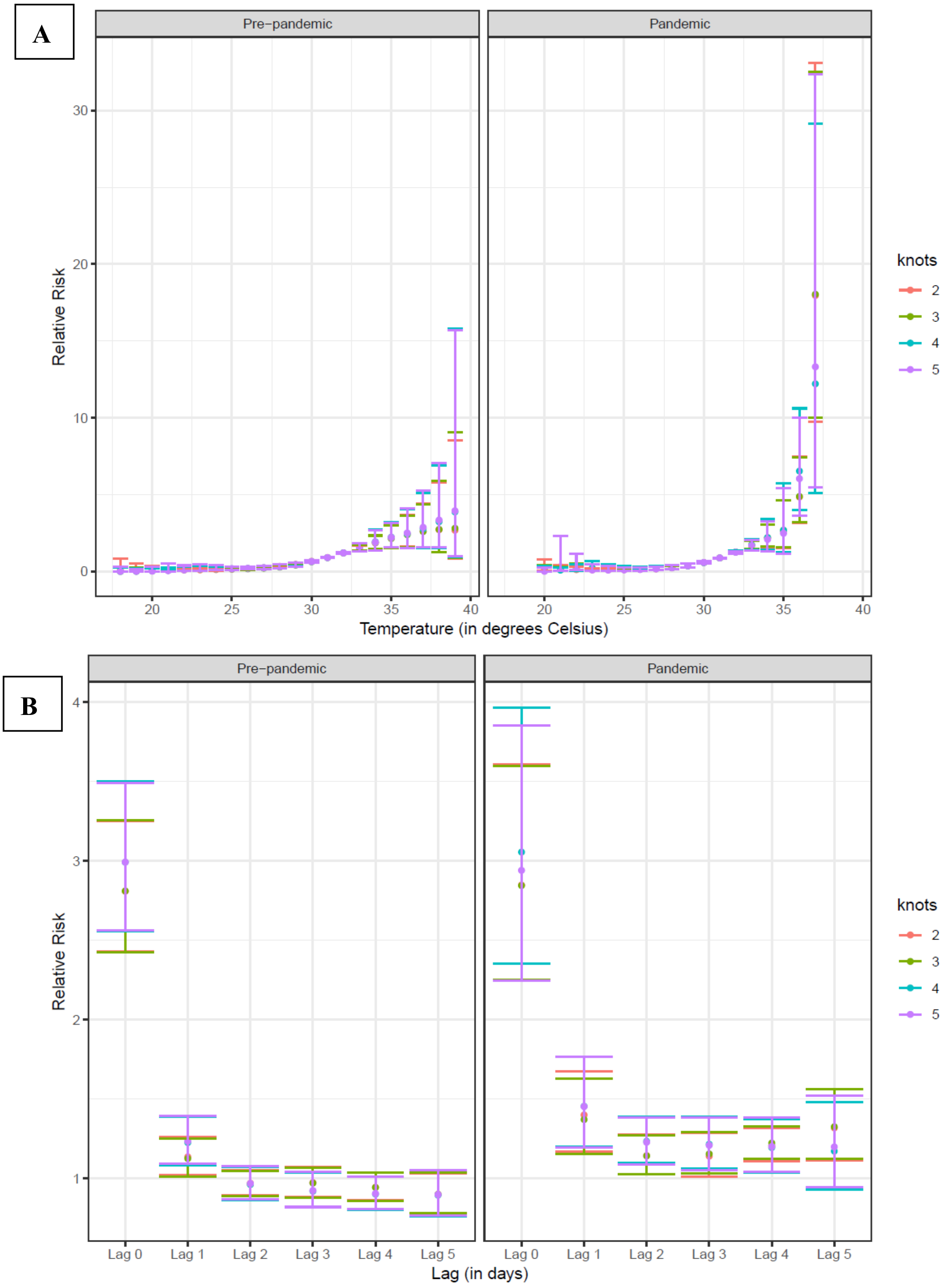

Here, we varied the number of knots from 2 to 5 (represented by orange, green, light blue, and violet colors, respectively) and examined whether the knot parameterization affected the maximum temperature-HIAT associations, for both pre-pandemic (*left panels*) and during pandemic (*right panels*) periods and in both exposure (*upper panels*) and lag (*lower panels*) dimensions.

Figure S9. Residual distribution accounting for seasonal patterns

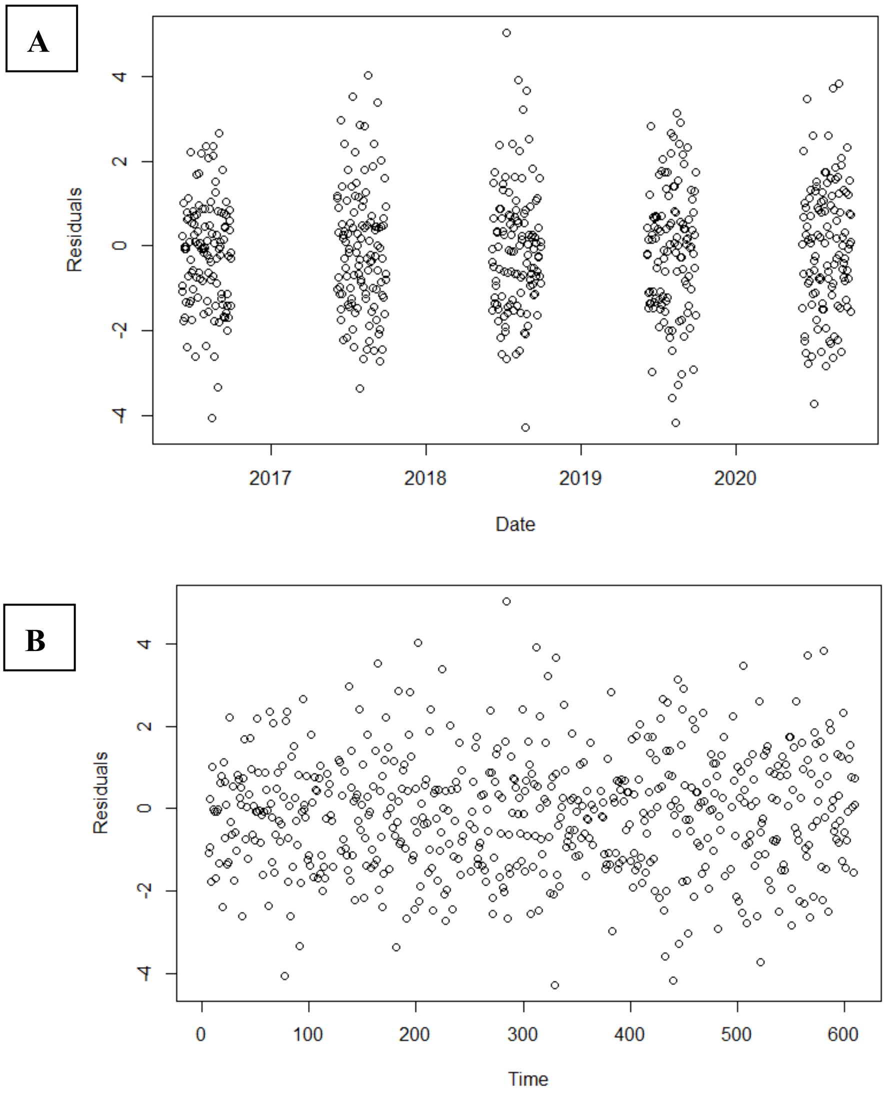

We observe a near homogenous distribution of the residuals across years, with no discernable pattern (*Panel A*). In order to make the distribution of the residuals more discernable across the time period of observation, we used the continuous counter time instead of the day of the season (*Panel B*). We noted a similar non-discernable pattern in the residuals across the observation time period.
